# A metabolomic analytical approach permits identification of urinary biomarkers for *Plasmodium falciparum* infection: a case–control study

**DOI:** 10.1186/s12936-017-1875-z

**Published:** 2017-05-30

**Authors:** Salah Abdelrazig, Catharine A. Ortori, Gail Davey, Wakgari Deressa, Dhaba Mulleta, David A. Barrett, Alemayehu Amberbir, Andrew W. Fogarty

**Affiliations:** 1 Centre for Analytical Bioscience, School of Pharmacy, University of Nottingham, Nottingham, NG7 2RD UK; 2 Wellcome Trust Centre for Global Health Research, Brighton and Sussex Medical School, Brighton, UK; 3 Department of Preventive Medicine, School of Public Health, College of Health Sciences, Addis Ababa University, Addis Ababa, Ethiopia; 4 East Shewa Zone Health Department, Oromia Regional State, Adama, Ethiopia; 5 School of Medicine, University of Nottingham, Nottingham, UK

**Keywords:** Malaria, Urine, Falciparum, Biomarker, Metabolomics

## Abstract

**Background:**

Currently available diagnostic techniques of *Plasmodium falciparum* infection are not optimal for non-invasive, population-based screening for malaria. It was hypothesized that a mass spectrometry-based metabolomics approach could identify urinary biomarkers of falciparum malaria.

**Methods:**

The study used a case–control design, with cases consisting of 21 adults in central Ethiopia with a diagnosis of *P. falciparum* infection confirmed with microscopy, and 25 controls of adults with negative blood smears for malaria matched on age and sex. Urinary samples were collected from these individuals during presentation at the clinic, and a second sample was collected from both cases and controls 4 weeks later, after the cases had received anti-malarial medication. The urine samples were screened for small molecule urinary biomarkers, using mass spectrometry-based metabolomics analyses followed by multivariate analysis using principal component analysis and orthogonal partial least square-discriminant analysis. The chemical identity of statistically significant malaria biomarkers was confirmed using tandem mass spectrometry.

**Results:**

The urinary metabolic profiles of cases with *P. falciparum* infection were distinct from healthy controls. After treatment with anti-malarial medication, the metabolomic profile of cases resembled that of healthy controls. Significantly altered levels of 29 urinary metabolites were found. Elevated levels of urinary pipecolic acid, taurine, N-acetylspermidine, N-acetylputrescine and 1,3-diacetylpropane were identified as potential biomarkers of falciparum malaria.

**Conclusion:**

The urinary biomarkers of malaria identified have potential for the development of non-invasive and rapid diagnostic test of *P. falciparum* infection.

**Electronic supplementary material:**

The online version of this article (doi:10.1186/s12936-017-1875-z) contains supplementary material, which is available to authorized users.

## Background

Malaria is endemic in 104 tropical and subtropical countries, and the most common cause of death from malaria in Africa is due to infection with *Plasmodium falciparum* [1]. The World Health Organization (WHO) estimated that 3.3 billion people were at risk of being infected with malaria in 2013 [2]. Malaria is a curable disease if diagnosed early, but drug resistance has drastically increased in recent years especially for *P. falciparum* infections [3]. Although microscopy is considered to be the “gold standard” for the diagnosis of malaria, the method is invasive, time-consuming, and requires expert skills. Rapid diagnostic tests (RDTs) [4, 5] have facilitated early diagnosis of malaria, but still require blood samples that may delay presentation, particularly in areas with high prevalence of Human Immunodeficiency Virus (HIV) infection [6, 7].

There are no studies of the use of metabolomics to identify urinary biomarkers for *P. falciparum* infection. As urine samples are readily available and do not require venepuncture, they have potential as a non-invasive approach for the early diagnosis of *P. falciparum* infection. A case–control study design was used to identify novel urinary biomarkers for *P. falciparum* infection using metabolomic methodology, and to explore if these biomarkers return to normal after treatment with anti-malarial medication.

## Methods

### Study population

The study used a case–control design. Cases were adults diagnosed with *P. falciparum* infection using blood-film microscopy at Adama Malaria Control Laboratory Centre in East Shewa Zone of Oromia Regional State in Ethiopia, from September to November 2013. Urine samples were collected from all cases at baseline (PF1) and again 4 weeks after they had received treatment with anti-malarial medication (PF2). Controls were healthy sex-matched adults who were a similar age to cases and had negative blood films for malaria parasites. Urine samples were also collected from controls at baseline (C1) and again after 4 weeks (C2). All participants provided informed consent. Ethical approval for the study was obtained from the Ethiopian Ministry of Science and Technology, the Institutional Review Board of the College of Health Sciences, Addis Ababa University and University of Nottingham Ethics Committees. Collecting samples on 20 cases and 20 controls would give 80% power to detect a one standard deviation difference in biomarkers between cases infected with malaria and convalescent samples/healthy controls.

### Urine sample collection, transport and storage

All urine samples were collected in urinary collection vessels without the use of preservatives and kept at −20 °C. After transport to UK, samples were aliquoted into cryotubes (6 × 1.0 mL) and stored in a −80 °C freezer. Simple urinalysis was performed to check for unwanted contaminated by haemolysis in the study samples using reagent strips (SureScreen Diagnostics, Derby, UK).

### Metabolomic analysis of samples

The urine samples were analysed in 60 µL aliquots using ultra-high performance liquid chromatography coupled to high resolution mass spectrometry (UHPLC-HRMS) using the protocol detailed in Additional file 1. All samples were analysed in a single analytical run with inclusion of pooled quality control (QC) samples. The chemical identity of selected urinary metabolites was confirmed by fragmentation analysis using ion-trap mass spectrometry and comparison with authentic standards (Additional file 1).

### Data analysis and metabolite identification

The raw data from UHPLC-HRMS analysis were acquired and visualized with Xcalibur v2.1 software (Thermo Scientific, USA). The performance of the analytical method was validated by monitoring a representative set of 60 urine metabolites in the pooled quality control sample for retention time shifts, mass accuracy and relative standard deviations (RSD %) of peak areas. For the metabolomics analysis, datasets from malaria patients and healthy controls were pre-processed using Progenesis QI software (Nonlinear Dynamics, Newcastle, UK) for peak picking, peak alignments, normalization and peak deconvolution. In addition, the quality of the datasets obtained from the LC–MS analysis was assessed against acceptance criteria in a standardized metabolomics approach [8].

The initial analysis compared urinary biomarker levels between cases with *P. falciparum* infection and healthy controls, and subsequent analysis explored the impact of anti-malarial treatment and clinical recovery on the candidate biomarkers identified. Multivariate data analysis using principal component analysis (PCA) and partial least square-discriminant analysis (OPLS-DA) were used to investigate possible metabolic changes between all classes in the study using Simca P +14 (MKS, Umeå, Sweden). The resultant OPLS-DA models were validated using cross-validation, permutation test and prediction method based on randomly selected training (50%) and test sets (50%) of samples. The specificity and selectivity of the prediction models were tested using area under the ROC (receiver operating characteristic) curve (AUC).

### Tentative identification of urinary biomarkers of malaria

Metabolites responsible for the classification between falciparum malaria patients (PF1) and healthy controls (C1) were selected according to the variable importance for the projection (VIP) values from the OPLS-DA models. Metabolites with VIP score more than 1.0 were chosen and an ArcSinh transformation was applied to restore normality. The selected metabolite intensities across malaria patients’ and healthy controls’ samples were subjected to the Student’s *t* test and the generated p values were adjusted using false discovery rate to account for the multiple comparison problem. Top metabolites that differed significantly (q ≤ 0.05) between case and control groups were selected and tentative identification of malaria biomarkers was achieved by interrogating the Urine Metabolome Database, (http://www.urinemetabolome.ca), using accurate mass measurements within 5 ppm mass error. Confirmation of identity of some biomarkers was performed by means of fragmentation analysis using ion-trap MS and comparison with authentic standards.

## Results

21 individuals with *P. falciparum* infection demonstrated on microscopic examination of blood films and 25 healthy adults with blood films negative for malaria parasites provided urinary samples for analysis. The mean age of the cases was 30.7 years [standard deviation (sd) 10.3, range 17–50] and 17 (81%) were male. The mean age for the controls was 32.1 years (sd 10.6, range 19–54) and 20 (80%) were male. The validation of the LC–MS performance for metabolomics analysis is presented in Additional file 1, and demonstrated that a consistent and stable metabolomics analysis was achieved for the malaria urine samples. PCA analysis of the entire data set showed consistent clustering of QC samples (Additional file 2: Figure S1).

### Cross-sectional urinary metabolomics analysis of malaria cases and healthy controls

**Fig. 1:**
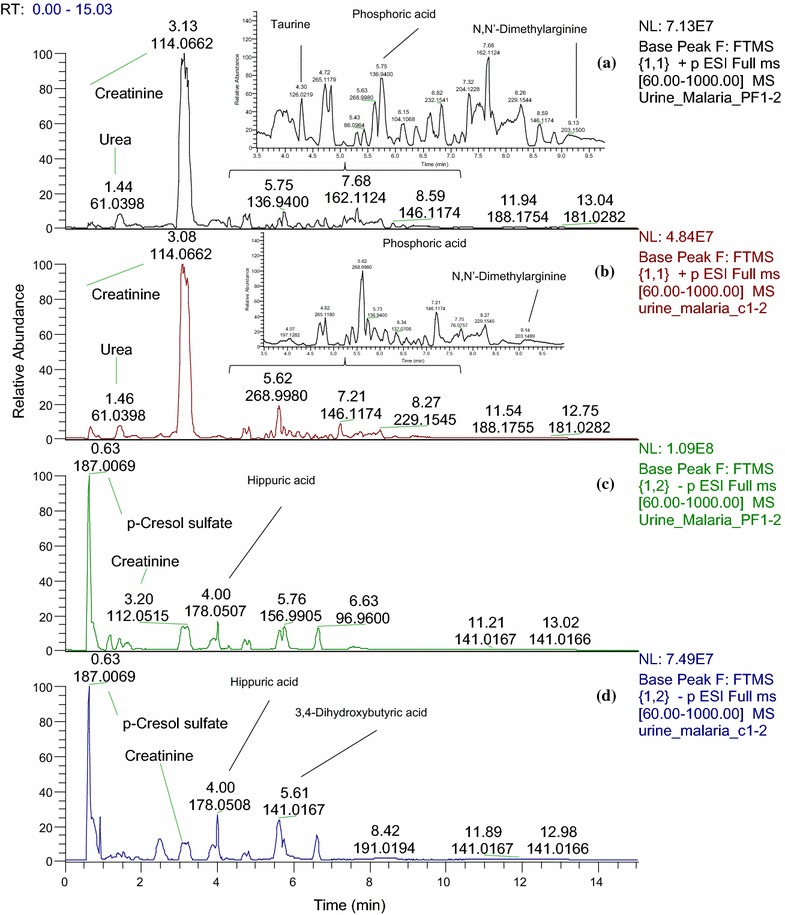
UHPLC-HRMS base peak chromatograms (BPC) obtained from malaria and control urine samples. BPC of **a** malaria patients (ESI+), **b** healthy controls (ESI+), **c** malaria patients (ESI−) and **d** healthy controls (ESI−) analysed using a HILIC column. Amino acids such as l-alanine, l-tryptophan, tyrosine and phenylalanine were eluted within the retention time range of 5–6.5 min, whereas, organic acids such as 4-aminohippuric acid, homovanillic acid, lactic acid, uric acid and 2-hydroxyisobutyric acid were detected within a wider retention time window (0.5–5 min). Some urinary pyrimidine nucleosides such as cytidine and uridine were eluted within 2.5 min

**Fig. 2:**
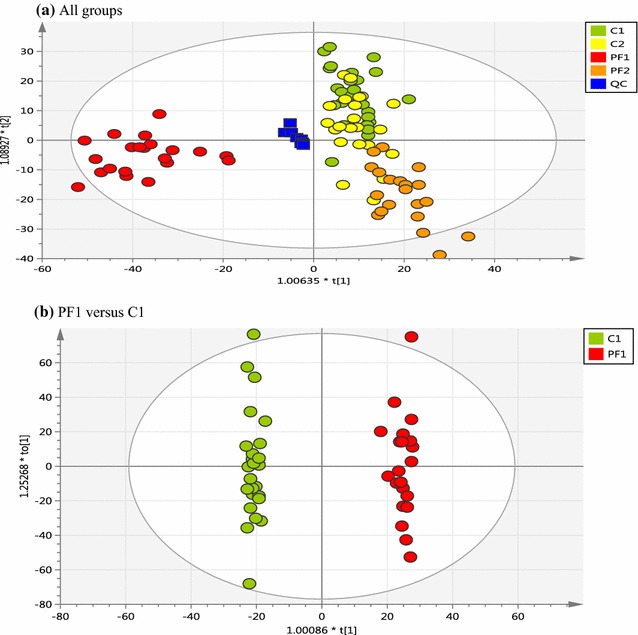
OPLS-DA score plots obtained from urinary metabolomic analysis of malaria patients and controls. **a** OPLS-DA model was built using control samples (C1; baseline, *green circles*, n = 25), (C2; follow-up, *yellow circles*, n = 22), malaria samples: baseline (PF1; *red circles*, n = 21) and after antimalarial treatment (PF2; *light brown circles*, n = 20) and pooled QC (*dark blue squares*), **b** presents OPLS-DA model generated from C1 and PF1 samples

Typical LC–MS base peak chromatograms obtained from urine samples of malaria patients and healthy controls are shown in Fig. 1. Adequate chromatographic separation was attained with most of metabolite peaks eluted within 9 min. The metabolites observed in the chromatograms mainly comprised a range of organic acids, amino acids and pyrimidine nucleosides. The metabolomics datasets generated a large number of analytical variables (Table 1) which were subjected to multivariate data analysis to classify the urinary metabolites contributing to the separation of the clinical groups in the study. OPLS-DA analysis showed separation and clustering of malaria samples (PF1) from the rest of the groups with no clear differences in urine metabolic profiles between the samples from the two control groups (C1 and C2) with the convalescent samples from the treated cases collected after 4 weeks (PF2) (Fig. 2). The clustering of malaria samples after 4 weeks (PF2) within the control groups region in the models indicates no significant differences between those samples.

**Table 1:** Multivariate analysis and validation of OPLS-DA models of malaria patients and healthy controls

Description	LC-MS
Peak detected	
ESI+	6278
ESI−	3466
ESI+ and ESI−	9744
Cross-validation	
R^2^Y	0.993
Q^2^	0.583
Permutation test	
Intercept^a^	−0.268
External validation: classification (training/test models)	
Sensitivity (%) (true positive rate (TPR) = TP/(TP + FP)	80%
Specificity (%) (true negative rate (TNR) = TN/(TN + FN)	77%
Accuracy (%) = (TP + TN)/(TP + FP + TN + FN)	78%
ROC curve (AUC)^b^ (TPR vs FPR)	0.83
Classification based on a selected set of biomarkers^c^	
Sensitivity (%)	91%
Specificity (%)	91%
Accuracy (%)	91%
ROC curve for individual set of metabolites	0.92

^a^The model is considered valid when the regression line of the permuted Q^2^ values intercept at, or below zero

^b^AUC: Area under receiver operating characteristic curve, which is the total area under the curve of sensitivity “true positive rate (TPR)” vs 1-specificity “false positive rate (FPR)”, ideal model gives AUC = 1

^c^Selected set of biomarkers were 1,3 diacetyl propane, 2-octanedioic acid, N-prolyl histidine, taurine, N-acetylputrescine, N-acetylasparagine, N-acetylspermidine and N-acetylglutamine

Subsequent OPLS-DA models were obtained from malaria patients (PF1) and healthy controls (C1) datasets only, total separation between the two groups were observed (Fig. 2). The OPLS-DA model was evaluated using cross-validation. The R^2^Y and Q^2^ values were R^2^Y = 0.993 and Q^2^ = 0.583 (Table 1). A successive permutation test was carried out for the OPLS-DA model to test if the good predictive ability of the model was due to data overfitting. The Q^2^-intercept values of the regression lines of the Y-permuted Q^2^ values were less than the Q^2^ values of the tested OPLS-DA models, and intercepted at −0.268, indicating a reliable predictive power of the generated model which was not due to data overfitting (Table 1). In addition, rigorous testing of the classification performance of the OPLS-DA model was performed using prediction models. The predictive accuracy, sensitivity and specificity of the OPLS-DA model was 78, 80 and 77% (Table 1), indicating a reliable and comparable predictive power of the model. The robustness of the generated OPLS-DA model was further validated using an unbiased approach, the area under the ROC curve (AUC) [9]. The OPLS-DA models gave comparable results to those obtained using training/test sets with AUC of 0.83, indicating that the predictability of the models was robust and valid.

A list of urinary metabolites which make a major contribution to the predictive OPLS-DA model between malaria patients and healthy controls is shown in Table 2. The fold change between malaria patients and controls is indicated, together with the false discovery rate (FDR) and an indication of the level of structural confirmation of each metabolite. Examples of confirmation of metabolites structures by LC–MS/MS are given in Additional file 2: Figures S2 and S3. A further ROC curve based on eight main biomarkers including 1,3 diacetyl propane, 2-octanedioic acid, N-prolyl histidine, taurine, N-acetylputrescine, N-acetylasparagine, N-acetylspermidine and N-acetylglutamine was generated. The predictive accuracy, sensitivity and specificity of these biomarkers were 91, 91 and 91%, respectively, with AUC of 0.92 (Table 1), indicating that these biomarkers have the potential to serve as urinary biomarkers of malaria.

**Table 2:** Tentative identification of urinary biomarkers showing differences between malaria patients and healthy controls using UHPLC-HRMS

Metabolite MW (Da)	Tentative identification	HMDB ID	Formula	RT (min)	q value	Fold change	Pathway/process	Confirmation of identity
60.0325	Urea	HMDB00294	CH_4_N_2_O	1.45	2.51E−05	1.70	Arginine and proline metabolism, urea cycle [1, 2]	(a)
76.0160	Glycolic acid	HMDB03035	C_2_H_4_O_2_	3.88	1.50E−02	1.34	Glyoxylate and dicarboxylate metabolism [1]	(a)
101.5395	Unknown	–	–	1.51	1.83E−04	8.03	–	–
113.0589	Creatinine	HMDB00562	C_4_H_7_N_3_O	3.11	1.43E−02	1.13	Arginine and proline metabolism [1]	(a, c)
118.0266	Succinic acid	HMDB00254	C_4_H_6_O_4_	3.08	4.99E−02	1.08	Tricarboxylic acid cycle, Propanoate metabolism [1, 2]	(a, c)
119.0582	l-Threonine	HMDB00167	C_4_H_9_NO_3_	6.53	4.08E−04	3.02	Aminoacyl-tRNA biosynthesis [1, 2]	(a, c)
125.0147	Taurine	HMDB00251	C_2_H_7_NO_3_S	4.29	2.96E−05	5.39	Taurine metabolism, Nitrogen metabolism [1]	(a, c)
128.0837	1,3-Diacetylpropane	HMDB29165	C_7_H_12_O_2_	6.46	7.62E−04	35.89	Polyamine metabolism [2]	(a)
129.0790	Pipecolic acid	HMDB00070	C_6_H_11_NO_2_	2.18	3.28E−02	1.34	Lysine degradation [1]	(a)
130.1106	N-Acetylputrescine	HMDB02064	C_6_H_14_N_2_O	6.62	3.23E−04	1.69	Arginine and proline metabolism [1, 2]	(a, b)
139.0633	3,4-Dihydroxybenzylamine	HMDB12153	C_7_H_9_NO_2_	3.25	7.57E−04	2.00	Not available	(a)
162.0528	3-Hydroxyadipic acid	HMDB00345	C_6_H_10_O_5_	1.50	2.66E−05	3.40	Fatty acid metabolism [1]	(a)
172.0736	2-Octenedioic acid	HMDB00341	C_8_H_12_O_4_	6.46	1.52E−04	7.20	Fatty acid metabolism [1]	(a, b)
174.1546	N-Acetylasparagine	HMDB06028	C_6_H_10_N_2_O_4_	3.61	7.33E−06	2.27	Asparagine catabolism [1]	(a)
187.1685	N-Acetylspermidine	HMDB01276	C_9_H_21_N_3_O	11.90	9.18E−05	1.72	Polyamine metabolism [1, 2]	(a, b)
188.0797	N-Acetylglutamine	HMDB06029	C_7_H_12_N_2_O_4_	3.59	2.30E−04	3.33	Not available	(a)
188.1525	Trimethyl-l-lysine	HMDB01325	C_9_H_20_N_2_O_2_	7.41	9.44E−07	2.60	Carnitine biosynthesis [1]	(a)
195.0532	3-Hydroxyhippuric acid	HMDB06116	C_9_H_9_NO_4_	4.05	2.05E−03	-3.00	Fatty acid metabolism [1]	(a)
208.0955	Unknown	–	–	6.80	1.46E−03	4.44	–	
209.0434	Unknown	–	–	1.41	3.80E−07	2.18	–	
210.0528	Vanilpyruvic acid	HMDB11714	C_10_H_10_O_5_	1.46	1.56E−03	4.36	Vanilactic acid biosynthesis [1]	(a)
212.0794	Unknown	–	–	3.88	1.43E−05	3.36	–	–
217.1063	Alanyl-glutamine	HMDB28685	C_8_H_15_N_3_O_4_	6.87	2.45E−05	5.45	Protein catabolism [1]	(a)
244.0694	Uridine	HMDB00296	C_9_H_12_N_2_O_6_	1.64	6.83E−07	1.63	Pyrimidine metabolism [1, 2]	(a, c)
252.1222	Prolyl-Histidine	HMDB29019	C_11_H_16_N_4_O_3_	2.93	8.29E−05	5.86	Protein catabolism [1]	(a)
268.0808	Inosine	HMDB00195	C_10_H_12_N_4_O_5_	1.42	2.17E−06	2.62	Purine metabolism [1, 2]	(a, c)
281.1124	1-Methyladenosine	HMDB03331	C_11_H_15_N_5_O_4_	5.68	8.07E−05	1.51	Not available	(a)
282.0961	1-Methylinosine	HMDB02721	C_11_H_14_N_4_O_5_	1.78	1.26E−03	2.15	Not available	(a)
285.0961	N4-Acetylcytidine	HMDB05923	C_11_H_15_N_3_O_6_	1.56	5.76E−05	3.37	Degradation of transfer ribonucleic acid (tRNA) [1]	(a)

*RT* retention time, *MW* molecular weight, *q value* is the adjusted Student’s t test p value using false discovery rate (FDR), the positive value of fold change means a higher level of metabolite in malaria patients compared to healthy controls, whereas the negative value represents a lower level of metabolite. Pathway existence: [1] human and [2] *P. falciparum*. The identity of biomarkers was confirmed using (a) exact mass and/or RT matched with database, (b) MS/MS spectra matched with reference spectra and/or (c) MS/MS spectra matched with spectra of authentic standards

## Discussion

This is the first study to use a metabolomics approach to identify urinary biomarkers for *P. falciparum* infection in humans. The analysis clearly identified a number of candidate biomarkers that are elevated in individuals with active infection confirmed by blood-smear microscopy. Levels of these molecules decrease after treatment with anti-malarial medication, suggesting that these molecules are biomarkers of active infection.

The strengths of these data include the prospective testing of the hypothesis that a metabolomics approach can identify biomarkers for *P. falciparum* infection in humans. A case–control study design was used, with prospective data collection in cases after they were treated and had recovered from the original infection, and also in controls. This allowed the candidate molecules identified in the cross-sectional study to be tested for their response to treatment, and hence reduced the possibility of false-positive outcomes as a consequence of multiple hypotheses testing that is a concern with this type of statistical analysis. However, these observations are preliminary and require confirmation in other datasets, before we can be confident that these associations are causal and these molecules are clinically useful biomarkers for infection with *P. falciparum* infection.

The increased level of succinic acid, taurine, alanine and pipecolic acid in malaria patients was consistent with previously reported studies [10–14], but the altered level of metabolites such as 1,3-diacetylpropane, N-acetylspermidine and N-acetylputrescine in the urine of malaria patients compared to healthy controls was observed for the first time, suggesting that these may be urinary biomarkers of malaria. In *P. falciparum* infection, there is a constant dynamic metabolic interplay between the host and the parasite during the course of infection that may perturb the biochemical profiles of both the parasite and the host. The parasite invasion induces a constellation of responses by the host which are collectively known as “active-phase responses” [15]. This phase is characterized by metabolic, immunological, neuro-endocrine and behavioural alterations to the host [16]. Hence, the altered level of metabolites observed in malaria patients compared to healthy controls might be a direct signal of parasite activity (parasite-specific metabolites) or be the consequence of the host response to the effect of the parasite on different organs during the acute phase of infection. Moreover, during the course of infection the parasite releases certain metabolites which induce the host metabolic response, so metabolites of parasite-specific molecules may accumulate in different body fluids. The metabolites directly related to the parasite are good biomarker candidates of the infection; however, their altered levels in different body fluids depends on the level of parasitaemia and the severity of the disease and they might not be detected in the early stages of the disease [17].

An increased level of alanine was observed in malaria patients compared to healthy controls, suggesting that lactic acid was converted to alanine, suggesting evidence of enhanced glycolysis pathway activity during the course of infection, consistent with recent observations [17]. However, alanine is also an essential precursor for gluconeogenesis in the liver and an elevated level may also be an indication of impaired hepatic gluconeogenesis or perturbed amino acid metabolism as a result of hepatic dysfunction in malaria. The level of succinate, a human and a parasite tricarboxylic acid (TCA) cycle intermediate, was significantly elevated in the urine of malaria patients compared to healthy controls, indicating enhanced metabolic TCA cycle activity by the parasite during the course of infection. The increased level of succinate in malaria patients may also indicate increased TCA cycle activity by the host to meet the increased energy demand caused by the infection, indicating perturbed energy metabolism in malaria. The increased level of succinate in *Plasmodium* infection was consistent with previous in vitro studies [18, 19]. Recently, Sengupta et al. reported an altered level of succinate in the urine of *P. vivax* infected patients [10, 11]. This result was consistent with the above finding, suggesting succinate is a potential urinary biomarker of malaria infection.

Abnormally high levels of pipecolic acid, trimethyl-l-lysine (methylated derivative of lysine), alanine, l-threonine, N-acetylglutamine (metabolite of glutamine) and N-acetylasparagine (metabolite of asparagine) were observed in the urine of malaria patients but not healthy controls. This finding was consistent with previously reported studies, in which abnormal levels of amino acids and amino acid metabolites were found in the urine and plasma of patients infected with *P. falciparum* [17]. A high level of taurine (a sulphur amino acid) was also observed in the urine of malaria patients compared to healthy controls. Taurine is known to play an important role in the liver for detoxification of ammonia in individuals infected with malaria [20], suggesting that it may be up-regulated in the liver as a response to the increased body demand for ammonia elimination.

The increased excretion of urea in cases with *P. falciparum* infection is consistent with the observation that acute kidney injury occurs during malaria infection [21] and has also been reported elsewhere [17]. Significantly higher levels of acetylated polyamines such as 1,3-diacetylpropane, N-acetylspermidine and N-acetylputrescine were also found in the urine of malaria patients compared to healthy controls. This is the first time that altered levels of acetylated polyamines have been detected in the urine of malaria patients, and may provide potential surrogate biomarkers of malaria. The altered levels of 1,3-diacetylpropane, N-acetylspermidine and N-acetylputrescine in the urine of malaria patients suggest that excess putrescine and spermidine have been continuously detoxified by the body before excretion as a response to their excessive production by the parasite. Teng et al. [19] reported significantly elevated levels of putrescine and spermidine in *Plasmodium*-infected erythrocytes compared to non-infected ones.

## Conclusion

A metabolomics analysis of urine samples from a case–control study was used to identify possible diagnostic urinary biomarkers of *P. falciparum* infection. This approach identified a number of candidate molecules that are associated with the presence of and recovery from *P. falciparum* infection in human. This approach has the potential to lead to a non-invasive urinary diagnostic test for *P. falciparum* infection.

## Additional files

**Additional file 1.** Further methods.

**Additional file 2: Figure S1.** PCA score plots overview obtained from all malaria and control urine samples. **Figure S2.** MS/MS spectra comparison of tentitively identified taurine in the urine sample of malaria patients against its authentic standard. **Figure S3.** MS/MS spectra confirmation of tentitively identified succinic acid, creatinine, uridine and L-threonine in the urine sample of malaria patients against their authentic standards.
