# Supplementary material for: A metabolomic analytical approach permits identification of urinary biomarkers for Plasmodium falciparum infection: a case–control study
Source: Malar J. 2017 May 30;16:229. doi: 10.1186/s12936-017-1875-z (PMC5450092; doi:10.1186/s12936-017-1875-z)
Supplement: Supplementary file 1 — Additional file 1. Further methods. [file 12936_2017_1875_MOESM1_ESM.docx]

***Materials and reagents***

LC-MS grade ammonium acetate was supplied from Fluka, Sigma-Aldrich, Netherlands. Water (VWR international, EC), formic acid, 0.1% formic acid in water (Sigma-Aldrich, Germany), acetonitrile and methanol (Fisher Scientific, UK) are MS grade and minimum handlings were performed to minimise any possible contamination. Inosine, taurine, succinic acid, urea and uridine were purchased from Sigma-Aldrich, Germany. Creatinine was purchased from Alfa Aesar, UK, whereas L-threonine was from Acros Organics, USA.

## *Preparation of urine samples and authentic standards*

35 metabolites were selected to represent a range of metabolite structures naturally found or expected in healthy human urine [1, 2]. A mixture of these compounds was prepared in MS grade water in the concentration range of 2.7-67.8 µg/mL as “artificial urine”. Artificial urine was used to optimise LC-MS parameters and to check the analytical stability of the MS system during metabolomics analysis.

For LC-MS analysis, 60 µL aliquots of thawed urine were centrifuged at 10,000g for 10 min at 4˚C to remove particulates. 50 µL of the supernatant was added to 100 µL water in HPLC amber glass vials containing 200 µL micro glass inserts. A reagent blank was prepared following the same protocol without including urine.

For metabolomics analysis, a pooled QC sample was prepared by mixing 20 µL aliquots taken from each urine sample and treated the same as described for the study samples. Urine samples from malaria patients and healthy controls were randomised and analysed in a single analytical run using simultaneous positive and negative electrospray ionisation (ESI) modes for UHPLC-HRMS. Six injections of pooled QC sample were analysed at the beginning of the run to equilibrate the system prior the analysis. Pooled QC samples were interspaced throughout the run to check the stability, robustness, repeatability and performance of the analytical system.

## *LC-HRMS Analysis*

Chromatography was performed using Accela UPLC system (Thermo Fisher, USA) on BEH HILIC column (2.1 x 100 mm, 1.7 µm particle size, Waters, USA). The column was maintained at 40ºC and a flow rate of 400 µL/min. Mobile phases used were: (A) 50:50 and (B) 95:5 acetonitrile:ammonium acetate (10 mM final concentration). The gradient started with 1% (A) and increased to 100% (A) over 12 min then the composition was returned to its initial conditions and maintained for the second run (15 min total). The injection volume was 5 µL and samples were maintained at 4 ºC during the analysis. An Orbital trap mass spectrometer (Exactive-Orbitrap, Thermo Fisher Scientific, USA) was used in simultaneous ESI+ and ESI- modes for LC-MS. The operational parameters were: spray voltage 3.2 kV (ESI+), 2.4 (ESI-), capillary voltage 25 V (ESI+), -27 V (ESI-), sheath, auxiliary and sweep gas flow rate were: 20, 5 and 5 (arbitrary unit), respectively, for both modes. Capillary and heater temperature were maintained at 350 and 120ºC, respectively. Data were acquired in full scan mode with resolution 25,000 from *m/z* 60-1000 with 4 Hz scan rate.

## *LC-MS/MS analysis*

The identity of some urinary metabolites was confirmed using liquid chromatography-ion-trap mass spectrometry. An LTQ Velos mass spectrometer equipped with Accela UHPLC system (Thermo Fischer Scientific, USA) was used for fragmentation analysis. The LC and MS conditions were the same as those described for UHPLC-HRMS. Product ions were detected over a scanning range of *m/z* 50–1000. The MS/MS analysis carried out using data global mass list scanning mode with 35 eV collision energy and helium was used as the collision gas. Metabolite identification was confirmed by comparison with pure authentic standards.

## Validation of LC-HRMS performance for metabolomics analysis

The analytical performance of LC-MS for metabolomics analysis was evaluated using the data from quality control (QC) samples. In the pooled QC samples datasets the mean RSD% values of selected urine metabolites peak areas were 4.6 % (range: 0.1 - 12.1%). UHPLC-HRMS retention time shifts were less than 0.06 min (≤ 1% RSDs) and mass accuracy shift was less than 5 ppm in both positive and negative ion modes. The quality of the datasets obtained was assessed using all peaks present in at least 80% of the pooled QC samples. The RSD% across the mean peak areas was less than 30% for 73% of those peaks, which was lower than the recommended threshold for metabolomics analysis [[8](#_ENREF_8)]. Unsupervised PCA score plots of the sample sets showed adequate clustering of the QC samples towards the centre of the plots, as shown in Figure 1. These results validate the LC-MS analytical performance.

References

1. Brooks T, Keevil CW. A simple artificial urine for the growth of urinary pathogens. Lett Appl Microbiol. 1997;24:203-6.

2. Bouatra S, Aziat F, Mandal R, Guo AC, Wilson MR, Knox C, et al. The human urine metabolome. PLoS ONE. 2013;8:e73076.
