# Supplementary material for: A metabolomic analytical approach permits identification of urinary biomarkers for Plasmodium falciparum infection: a case–control study
Source: Malar J. 2017 May 30;16:229. doi: 10.1186/s12936-017-1875-z (PMC5450092; doi:10.1186/s12936-017-1875-z)
Supplement: Supplementary file 2 — Additional file 2: Figure S1. PCA score plots overview obtained from all malaria and control urine samples. Figure S2. MS/MS spectra comparison of tentitively identified taurine in the urine sample of malaria patients against its authentic standard. Figure S3. MS/MS spectra confirmation of tentitively identified succinic acid, creatinine, uridine and L-threonine in the urine sample of malaria patients against their authentic standards. [file 12936_2017_1875_MOESM2_ESM.docx]

**Figure S1**: PCA score plots overview obtained from all malaria and control urine samples. Control samples: Baseline (C1; green circles, n=25), Follow-up (C2; yellow circles, n=22), malaria samples: Baseline (PF1; red circles, n=21) and after anti-malarial treatment (PF2; light brown circles, n=20) and pooled QC (dark blue squares) analysed by LC-MS.

****

****

****

**Figure S2**: Example of an MS/MS spectra comparison of tentatively identified taurine in the urine samples of malaria patients against its authentic standard. The spectra present the matching of taurine fragment peaks (standard) against those obtained from metabolite in the sample (sample) confirms its chemical identity.

****

****

****

****

****

** **

****

****

****

****

****

****

**Figure S3:** MS/MS spectra confirmation of tentatively identified succinic acid, creatinine, uridine and L-threonine in the urine sample of malaria patients against their authentic standards. The spectra present the matching of standards fragment peaks (standard) against those obtained from the metabolites in the sample (sample).
